# A Materia Medica

**Published:** 1888-04

**Authors:** 


A MATERIA MEDICA. Containing Provings and Clinical Verifications of Nosodes and Morbific Products. By Samuel Swan, M. D. New York. 1888.

As every one knows, Dr. Swan has for many years been interested in the study of these "Morbific Products." Some of them are doubtless very useful remedies, and when well proven will serve the prescriber well. In this "fascicle" the clinical and pathogenetic symptoms of Saccharum Lactis and of Lac caninum are given. Of Saccharum we know but little, having never used it. Of Lac can. we can say it is a most useful remedy. In this volume Lac caninum is given more completely than ever before. It is arranged by Dr. Berridge, a diligent and reliable worker in the field of Materia Medica. Many comparisons of other drugs by the late Dr. Ad. Lippe are given and enhance the value of the work.

A few days before reviewing this volume we prescribed Lac-can. for a violent headache, coming on in spells, first in one eye, next in the other, and then again in the first eye. The pain constantly changed from eye to eye, was very severe, worse from motion, etc. Now, on page 53, second symptom, we find our case well described. Other remedies have failed, and we hope to be able to report another triumph for Lac-can.

It is to be hoped that Dr. Swan will meet with sufficient encouragement to enable him to continue his *Materia Medica*. The price of this fascicle is, we believe, only seventy-five cents. No one should be without it who desires to know the uses of a most valuable remedy.

